# Concentrations, Deposition, and Effects of Nitrogenous Pollutants in Selected California Ecosystems

**DOI:** 10.1100/tsw.2001.395

**Published:** 2001-11-28

**Authors:** Andrzej Bytnerowicz, Pamela E. Padgett, Sally D. Parry, Mark E. Fenn, Michael J. Arbaugh

**Affiliations:** USDA Forest Service, Pacific Southest research Station, Riverside, CA 92507, USA

**Keywords:** nitrogen, air pollutants, forests, coastal sage, foliar damage

## Abstract

Atmospheric deposition of nitrogen (N) in California ecosystems is ecologically significant and highly variable, ranging from about 1 to 45 kg/ha/year. The lowest ambient concentrations and deposition values are found in the eastern and northern parts of the Sierra Nevada Mountains and the highest in parts of the San Bernardino and San Gabriel Mountains that are most exposed to the Los Angeles air pollution plume. In the Sierra Nevada Mountains, N is deposited mostly in precipitation, although dry deposition may also provide substantial amounts of N. On the western slopes of the Sierra Nevada, the majority of airborne N is in reduced forms as ammonia (NH_3_) and particulate ammonium (NH_4_) from agricultural activities in the California Central Valley. In southern California, most of the N air pollution is in oxidized forms as nitrogen oxides (NO_x_), nitric acid (HNO_3_), and particulate nitrate (NO_3_) resulting from fossil fuel combustion and subsequent complex photochemical reactions. In southern California, dry deposition of gases and particles provides most (up to 95%) of the atmospheric N to forests and other ecosystems. In the mixed-conifer forest zone, elevated deposition of N may initially benefit growth of vegetation, but chronic effects may be expressed as deterioration of forest health and sustainability. HNO_3_ vapor alone has a potential for toxic effects causing damage of foliar surfaces of pines and oaks. In addition, dry deposition of predominantly HNO_3_ has lead to changes in vegetation composition and contamination of ground- and stream water where terrestrial N loading is high. Long-term, complex interactions between N deposition and other environmental stresses such as elevated ozone (O_3_), drought, insect infestations, fire suppression, or intensive land management practices may affect water quality and sustainability of California forests and other ecosystems.

## INTRODUCTION

Although ozone ($O_3$) is known as the air pollutant with the highest phytotoxic potential in California and other western states, nitrogenous (N) air pollutants significantly impact many sensitive western ecosystems. Serious effects may be expected from inorganic and organic nitrogenous pollutants in dry (gaseous, particulate) and wet (rain, fog, snow) forms. Direct toxic effects caused by nitric oxide (NO), nitrogen dioxide ($NO_2$), ammonia ($NH_3$) or peroxyacetyl nitrate (PAN) are possible at very high ambient concentrations such as those that have been observed in selected locations in Europe or Canada near fertilizer plants or in the immediate vicinity of accidental spillage of liquid $NH_3$[1]. In California, toxic effects of these pollutants are rather unlikely to occur at current ambient levels. In southern California in the 1960s and 1970s, however, toxic effects of PAN were documented for sensitive agricultural species, such as lettuce and tomatoes[2].

Recent evidence indicates that toxic effects can be expected at elevated concentrations of gaseous nitric acid ($HNO_3$). Phytotoxic responses have been shown for ponderosa pine and California black oak during short-term, elevated-level fumigations[3, 4]. Although toxic effects of $HNO_3$ have not yet been shown under ambient field conditions, new experimental data point out that this pollutant may pose serious problems to sensitive plants.

The long-term effects of N deposition have been shown to change ecosystem structure and function. Most of the knowledge on the subject has been gained from studies where wet deposition was the primary delivery source[5,6]. The effects of dry deposition are likely to result in similar responses; however, the chemical differences between vaporous and aqueous phase behavior of nitrogenous pollutants may result in differences in ecosystem responses. With a very high proportion of deposition occurring in the dry form over much of California, this area provides unique opportunities to study and understand dry deposition effects on ecosystem processes. Clearly, N dry deposition has resulted in excess available N in forests and other ecosystems[7], and this in turn may impact sustainability and biodiversity of the most sensitive ecosystems[8,9,10]. N saturation also results in contamination of ground- and surface waters from montane watersheds with excess nitrate[11].

In this article we provide information on concentrations of two ecologically important N air pollutants, $HNO_3$ vapor and $NH_3$, as well as N deposition to selected California ecosystems, and we show examples of deposition and toxic effects of $HNO_3$ on representative tree species of the mixed-conifer forest ecosystem.

## CONCENTRATIONS OF NITROGENOUS AIR POLLUTANTS

In Californian Mediterranean climate, about 80% of annual precipitation is deposited in the November to April period. This period coincides with predominately offshore breezes that transport pollutants generated in coastal communities westward over the Pacific Ocean rather than into inland valleys and mountains. During the dry summer months, heating of the landmasses in conjunction with the cool marine mass lead to predominantly onshore breezes during daylight hours and delivery of air pollutants to the western slopes of the Sierra Nevada, San Gabriel, and San Bernardino Mountain chains. The overriding influence of this climate on atmospheric deposition is the dominance of dry deposition over wet deposition throughout large areas of the state. The importance of dry deposition is greatest in areas where temperature inversions commonly occur, such as the Los Angeles basin[12]. In areas characterized by frequent high levels of urban photochemical smog, such as the Los Angeles and Sacramento air basins, oxidized N compounds, especially NO, $NO_2$, $HNO_3$ vapor, PAN, and particulate nitrate ($NO_3^-$), are the most common N compounds. In the areas adjacent to agricultural activities, such as the western slopes of the Sierra Nevada east of the agriculture-rich California Central Valley and the western San Bernardino Mountains located downwind of the Chino dairies, air pollutants containing reduced N compounds such as $NH_3$ and particulate ammonium ($NH_4^+$) dominate[12]. NO, $NO_2$, and PAN pollutants occur in high concentrations in the urban areas, but their concentrations in forests and wildlands are generally low and they do not typically pose immediate phytotoxic effects[1]. Contributions of these pollutants to terrestrial N loading are also small because deposition that is largely controlled by stomatal uptake and stomatal conductance is generally low during the periods of highest pollutant concentrations (the dry summer months). Concentrations of particulate $NO_3^-$ and $NH_4^+$ may be high in many urban areas, but because of their low deposition velocity they may be an important source of N in wildlands immediately adjacent to the source but relatively small contributors to deposited N at longer distances[13]. At the ambient concentrations occurring in California, particulate $NO_3^-$ and $NH_4^+$ do not seem to pose any direct toxic effects on plants.

Most of the information on N pollutant concentrations and N deposition in California ecosystems has been gathered for mixed-conifer forest ecosystems. These ecosystems are the most disturbed by human activities and management and among the most impacted by photochemical pollutants generated in rapidly developing urban areas of southern California, the San Francisco Bay Area, and the California Central Valley. Information on concentrations and deposition in these forests was reviewed[12], and a review of the effects of $O_3$ and N deposition on mixed-conifer forests in California recently has been published[14]. Since publication of these two papers, more data has been collected in Sequoia National Park of the western Sierra Nevada, Mammoth Mountain in the eastern Sierra Nevada, and the San Bernardino Mountains. Updated information on $HNO_3$ and $NH_3$ concentrations in several California ecosystems is presented in Tables 1 and 2. Less is known on NO and $NO_2$ concentrations in forest locations, but in general, hourly average concentrations of these pollutants do not exceed 31 and 94 mg/m$^3$, respectively[1]. Recent studies on elevational gradients in Sequoia National Park and the San Bernardino Mountains indicate that concentrations of $NO_2$, $NH_3$, and $HNO_3$ decrease with elevation and distance from the pollution source areas[15,16].

## ESTIMATED N DEPOSITION LOADING

Estimates of total N deposition to various ecosystems in California have been made[1,14,17,18,19]. These estimates are a sum of surface deposition measured with throughfall and branch-rinsing techniques; stomatal uptake of NO, $NO_2$, $HNO_3$, and $NH_3$; and wet deposition of $NO_3^-$ and $NH_4^+$ ions. These estimates show ~1 kg N/ha/year in the subalpine ecosystems of the eastern Sierra Nevada; ~1.5 kg N/ha/year in the subalpine ecosystems of the western Sierra Nevada; 2–4 kg N/ha/year in mixed-conifer forests of the northern Sierra Nevada; <10 kg N/ha/year in mixed-conifer forests of the western Sierra Nevada; 5–45 kg N/ha/year in mixed-conifer forests of southern California; and 20–35 kg N/ha/year in chaparral and coastal sage scrub ecosystems of southern California. For the mixed-conifer forest stands in the moder-

**TABLE 1**
**Summertime Concentrations of $HNO_3$ Vapor in California Ecosystems**

| Location and Type of Ecosystem | Period of Measurements | Concentrations (µg/m$^3$) | Reference |
|---|---|---|---|
| Eastern Brook Lake, subalpine | 1987 | 0.36 (24 h) | 31 |
| Mammoth Mountain, subalpine | 1999–2000 | 0.1–0.7 (24 h) | 32 |
| Whitaker Forest, mixed conifer | 1988–1990 | 1.1–1.8 (day); 0.3–0.7 (night) | 33 |
| Shirley Meadow, mixed conifer | 1989–1990 | 0.3–4.8 (day); 0.2–2.5 (night) | 34 |
| Sequoia National Park, mixed conifer | 1999 | 0.1–4.6 (24 h) | 15 |
| San Bernardino Mountains, mixed conifer/subalpine | 2000 | 1.9–5.1 (summer season) | 16 |
| Barton Flats, mixed conifer | 1992–1994 | 0.5–9.7 (24 h) | 13 |
| Tanbark Flats, chaparral | 1988–1991 | 3.0–27.3 (day); 0.4–4.5 (night) | 35 |
| Southern California, coastal sage | 1995–1996 | 3.5–22 (24 h) | 36 |

**TABLE 2**
**Summertime Concentrations of $NH_3$ in California Ecosystems**

| Location and Type of Ecosystem | Period of Measurements | Concentrations (µg/m$^3$) | Reference |
|---|---|---|---|
| Whitaker Forest, mixed conifer | 1988–1990 | 0.2–3.8 (day); 0.5–2.3 (night) | 33 |
| Shirley Meadow, mixed conifer | 1989–1990 | 0.8–5.5 (day); 0.3–4.7 (night) | 34 |
| Sequoia National Park, mixed conifer | 1999 | 0.8–9.4 (24 h) | 15 |
| San Bernardino Mountains, mixed conifer/subalpine | 2000 | 2.5–6.0 (summer season) | 16 |
| Barton Flats, mixed conifer | 1992–1994 | 0.6–2.6 (24 h) | 13 |
| Tanbark Flats, chaparral | 1988–1991 | 0.3–4.7 (day); 1.6–2.5 (night) | 35 |
| Southern California, coastal sage | 1995–1996 | 3.5–6 (24 h) | 36 |

ately polluted part of the San Bernardino Mountains, most of deposition is to plant surfaces, with only a small proportion taken up by the stomata (Fig. 1)[13]. Even in humid areas, dry deposition may be a significant contributor to terrestrial N loading. In southern California and other semiarid locations in the state, the majority of the deposited N, sometimes reaching 95% of total deposition, is in the dry form (Table 3). However, where fog incidence is a regular occurrence, deposition of fog is often a major N input pathway as well.

In the relatively open stands characteristic of the mixed-conifer forests of California, atmospheric deposition inputs are highly heterogenous. For example, estimates of total N deposition along four throughfall transects at Camp Paivika in the western San Bernardino Mountains (eight collectors per transect) ranged from 20–53 kg/ha/year, with inputs varying with amount of canopy cover[17]. Furthermore, during that particular study, nearly half of the N deposition was from dry deposition and half from wet deposition (fog + precipitation). In more recent studies, deposition inputs under mature ponderosa pine canopies at Camp Paivika over a 7-month period were 88–92 kg/ha, largely as a result of fog drip, while N deposition in open areas during this same time period was only 5.3 kg/ha[18]. These findings highlight the "hotspot" or heterogenous nature of deposition in these stands.

## NITRIC ACID VAPOR — AIR POLLUTANT OF A SPECIAL IMPORTANCE FOR SOUTHERN CALIFORNIA ECOSYSTEMS

$HNO_3$ may be the most ecologically important of the nitrogenous air pollutants in semiarid western ecosystems. Although ambient atmospheric concentrations of $HNO_3$ are typically lower than NO and $NO_2$, its greater importance to terrestrial ecosystems derives from the following characteristics: (1) it has a low retention time in the atmosphere and is rapidly deposited as vapor or in precipitation depending on environmental conditions[20]; (2) the vapor has a high affinity for many surfaces, typically reported as a "high deposition velocity"[21]; (3) $HNO_3$ is a strong oxidant and has the potential for attacking a variety of organic compounds[22]; and (4) it is a natural plant nutrient, readily absorbed and assimilated either directly by foliage or indirectly when it is incorporated into soils[21].

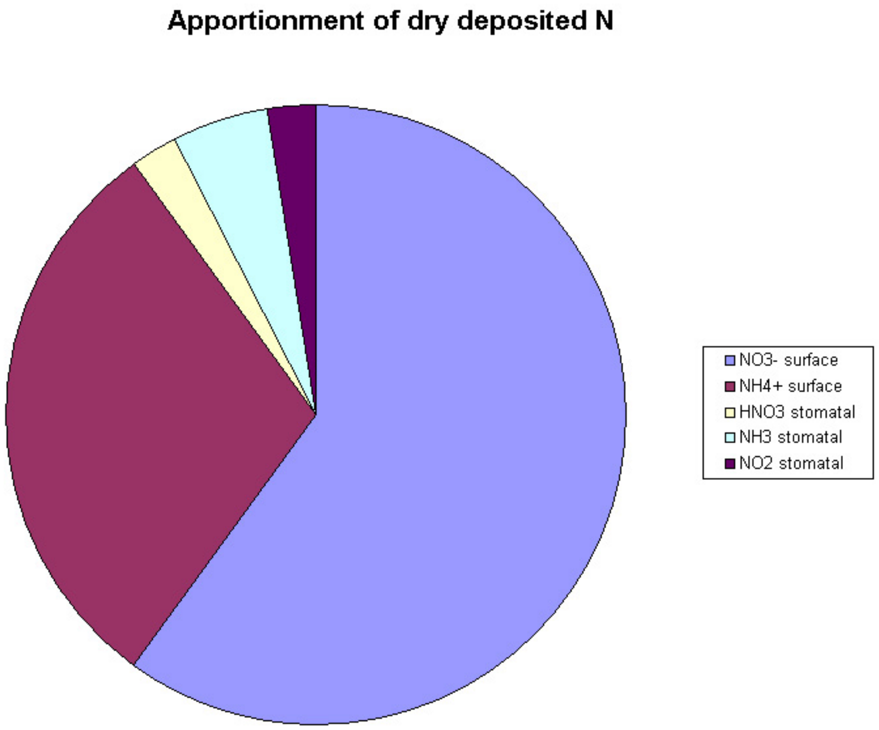

**FIGURE 1**. Apportionment of dry deposition to ponderosa pine foliage in summer 1993 at the moderately polluted site of the San Bernardino Mountains.

**TABLE 3**
**Estimated Percentage of Total Deposited N Due to Dry Deposition**

| % of Total N Deposition | Location or Region | Vegetation Type | Reference |
|---|---|---|---|
| 46 | North America | Temperate forests | 25 |
| 40 | Washington State | Douglas fir | 25 |
| 55 | Great Smokey Mountains | Conifer forest | 25 |
| 50 | Georgia | Conifer forest | 25 |
| 32–53 | Upper Midwest | Forest | 37 |
| 21–46 | Gulf states | Mixed forest | 37 |
| 31 | Southwestern Pennsylvania | Mixed forest | 38 |
| 16 | Maine | Mixed forest | 38 |
| 90–95 | Southern California | Costal sage scrub | 36 |
| 60–90 | Southern California | Mixed-conifer forest | 12 |

In the discussion below, two lines of evidence are presented to support the proposed significance of $HNO_3$ vapor as an ecological factor in semiarid ecosystems.

## Cuticular Degradation

Plant leaf surfaces are coated with a layer of epicuticular waxes generally consisting of organic compounds such as alkyl esters, aldehydes, and carboxylic acids, among others. Experimental evidence has shown that plants exposed to $HNO_3$ vapor at realistic doses, similar to those observed in the field, have produced characteristic changes on the foliar cuticular surfaces[3]. Significant changes in leaf surface characteristics that are consistent with oxidation processes have been identified using scanning electron microscopy to study leaves exposed to $HNO_3$. Physical perturbations to the cuticular wax surfaces of four tree species native to the mixed-conifer zone of western forests include formation of wax balls or droplets, alteration of rodlet and filamentous wax structures into plates and other amorphous shapes, development of lesions and cracks in the epicuticular surface, and blistering of the trichome surfaces[23].

Two examples are shown in Fig. 2. Two-year-old seedlings of ponderosa pine (*Pinus ponderosa*) and 3-year-old California

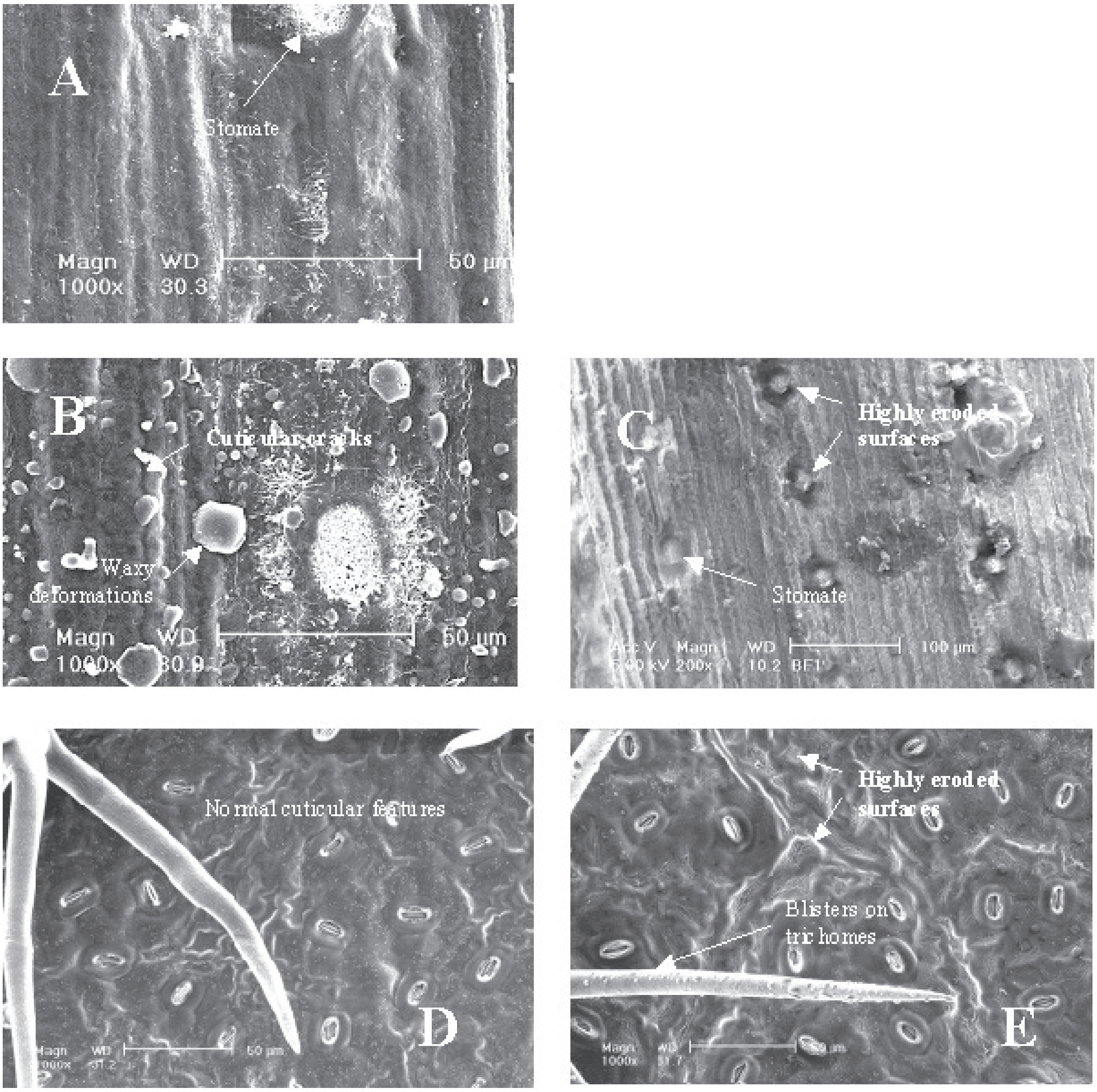

**FIGURE 2**. $HNO_3$ effects on plant cuticles: (A) ponderosa pine (*Pinus ponderosa*) needle prior to the $HNO_3$ exposure; (B) pine needle after 30 days of the $HNO_3$ exposure; (C) pine needle collected from the field location where $HNO_3$ levels are high; (D) California black oak (*Quercus kelloggii*) leaf prior to exposure; (E) oak leaf from the same plant after 24 days of the $HNO_3$ exposure (see text for details of the experiment).

black oak (*Quercus kelloggii*) seedlings were exposed in fumigation chambers to high (but realistic) levels of gaseous $HNO_3$. The surfaces of fully expanded needles prior to exposure are relatively smooth (Fig. 2A). After 4 weeks, the fumigated needles developed specific injury symptoms in the epicuticular wax surface. These changes intensified with increased $HNO_3$ exposure dose. After 30 days of exposure to high levels of $HNO_3$, waxy balls or granules appeared (Fig. 2B). Closer inspection of the surface beneath the new structures revealed that the waxy cuticular surface was missing and an electron-dense subsurface was exposed.

Needle samples collected from field sites with differing pollutant loads corroborated the experimental observations. The example shown in Fig. 2C is from the current year's foliage collected from a highly polluted site in the San Bernardino Mountains. The highly eroded surface, patches of missing cuticle, and the presence of waxy balls or globules are seen. The response of California black oak also demonstrated modification to leaf surfaces, but the injury symptoms differed from that of the pines, and the accumulation of new waxy structures such as balls or granules was less typical. The epicuticular surfaces of black oak leaves are typically smooth, sprinkled with very small waxy rodlets (Fig. 2D). Following $HNO_3$ fumigation, the cuticular surface became susceptible to cracking, and patches where the surface appeared eroded became more frequent with increasing dose (Fig. 2E).

The differential responses to $HNO_3$ vapor of these two plant species were consistent with differences of chemical composition of epicuticular waxes across genera. The extent of the changes in surface features was correlated with increased pollutant

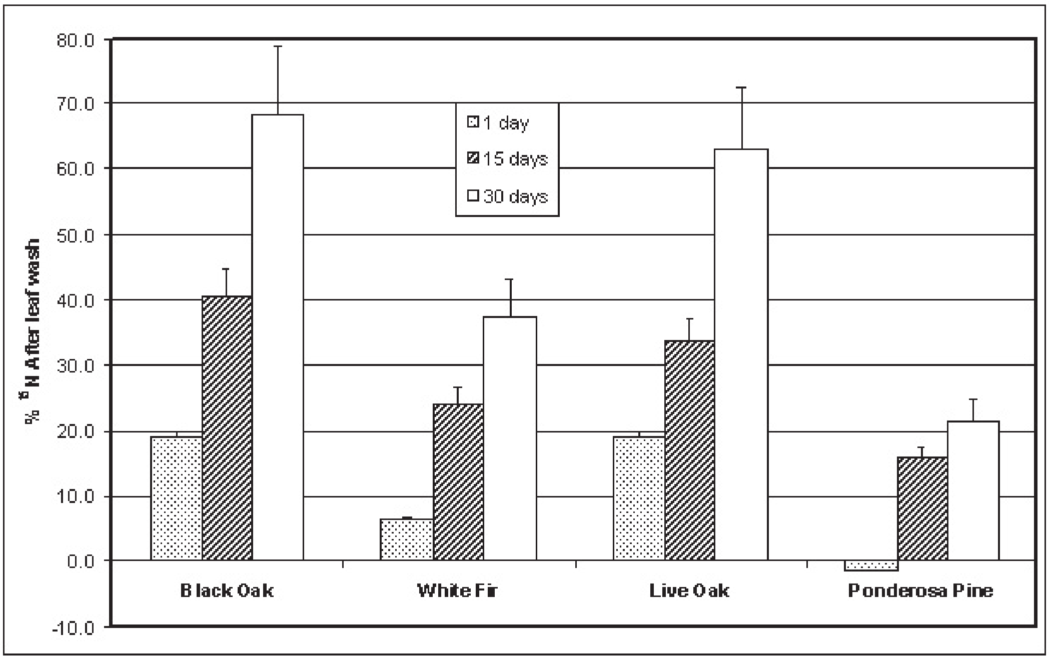

**FIGURE 3**. Percentage of $^{15}N$ from labeled $H^{15}NO_3$ remaining in leaves after washing with deionized water. Data present means after 1, 15, and 30 days exposures at elevated levels of the pollutant (for details refer to caption of Fig. 2).

dose[23]. Since the cuticle's primary function is to provide a physical barrier between the environment and the biologically active cells below, loss of epicuticular integrity could lead to increased losses of water and metabolites, increased susceptibility to insect or pathogen attacks, and greater opportunity for damage by other air pollutants such as $O_3$ or organic nitrogenous compounds[24].

## Foliar Absorption

A second line of experiments has investigated foliar absorption of dry-deposited $HNO_3$. The use of $^{15}N$-labeled $HNO_3$ has confirmed that atmospheric N is both bound to the surfaces of leaves and capable of penetrating the cuticle when stomata are closed[3,21]. Under field conditions, many throughfall studies conducted in forest stands demonstrate that deposited N is "consumed" in the canopy[7,25,26]. The magnitude of consumption is quite variable depending on species composition of the forest canopies, micrometeorological parameters, seasonal dynamics, and perhaps duration of exposure prior to measurement of deposition[7,25,26,27,28].

In controlled experiments, the proportion of $^{15}N$ retained by the foliage of four tree species increased with time of exposures (Fig. 3). This phenomenon indicates that with increasing pollutant dose, incorporation of $^{15}N$ was enhanced because of cuticular transport, stomatal uptake, or a combination of both processes. While all four species exhibited a similar pattern of $^{15}N$ assimilation, significant differences among the species were evident. For example, after 30 days of exposures, ponderosa pine retained less than 25% and California black oak and live oak more that 60% of the deposited $^{15}N$. Even after one day of exposure, the oak species retained nearly 20% of the $^{15}N$ deposited to the surfaces. Clearly foliar assimilation has the potential to strongly influence the nutrient balance in trees.

## CONCLUDING REMARKS

The effects of N deposition are insidious. It has been very difficult to prove direct cause-and-effect relationships between air pollution and ecological response in any ecosystem. However, in California ecosystems such as grasslands and shrublands, where plants are short lived and there is rapid turnover of individuals, the evidence is accumulating to implicate N deposition in shifts of ecological structure, function, and species composition[29,30]. While it seems unlikely that N deposition alone could cause measurable changes in forest ecosystems, clearly changes in the nutrient status of plants and chronic foliar injury would only serve to compound the natural and anthropogenic stresses already existing in California forests. In this context, long-term changes in physiology of trees, forest health and biodiversity, ecological processes, nutrient cycling, and water quality will be studied on the newly developing research network across the N-deposition and $O_3$-concentration gradients in the San Bernardino Mountains of southern California.

| | | |
|---|---|---|
| Received: | September | 4, 2001 |
| Revised: | October | 29, 2001 |
| Accepted: | November | 14, 2001 |
| Published: | November | 28, 2001 |

## BIOSKETCHES

**Andrzej Bytnerowicz** is an Ecologist with the USDA Forest Service, Pacific Southwest Forest and Range Experiment Station. He received a Ph.D. in Natural Sciences in 1981 from Silesian University in Katowice, Poland and an M.S. in Food Chemistry in 1972 from Warsaw Agricultural University, Poland. His research interests include development and tests of air pollution monitoring techniques for remote locations, development of models for air pollution distribution and deposition in mountain forest ecosystems, coordination of studies on effects of ozone and nitrogenous air pollutants in forests of California and Central Europe, investigations of effects of air pollutants on plant antioxidant systems.

**Pamela E. Padgett** is a Research Plant Physiologist with the USDA Forest Service, Pacific Southwest Forest and Range Experiment Station. She received a Ph.D. in Botany in December 1993 from the University of California, Riverside and a B.S. in Agronomy in September 1987 from Virginia Polytechnic Institute and State University, Blacksburg, Virginia. Her research interests are focused on understanding the fundamental mechanisms driving plant responses to air pollution and other anthropogenic disturbances. Current studies range from, laboratory studies of nitric acid (a common urban pollutant) uptake and assimilation by plant foliage, atmospheric deposition characteristics to soils and damage incurred by air pollutants on leaf cuticles, to field scale evaluations of industrial-generated dust on rare plants and habitats. The approach of much of this work is to apply biochemical and physiological tools to environmental problems.

**Michael J. Arbaugh** is a Forest Ecologist with the USDA Forest Service, Pacific Southwest Forest and Range Experiment Station. He received a B.S. in Biology from the University of California, Riverside in 1980, an M.S. in Statistics from the University of California, Riverside in 1984, and a Ph.D. in Forest Ecology from Colorado State University, Ft. Collins in 1995. His research interests include understanding the multiple air pollutant effects on ecosystems, including large-scale patterns of air pollution and air pollution injury to western forests, long-term changes in forest composition, and passive air pollution monitor development and application in Class I areas.

**Mark E. Fenn** is a Research Plant Pathologist with the USDA Forest Service, Pacific Southwest Research Station. He received a Ph.D. in Plant Pathology in 1986 from the University of California, Riverside and a B.Sc. in Agriculture in 1981 from University of Arizona, Tucson. His research interests include measurement of throughfall deposition, including a newly developed ion exchange “passive” throughfall collector for more extensive throughfall monitoring. Processing of nitrogen through the canopy, soil profile and throughout the watershed under varying nitrogen deposition loads is a major focus. The key role of nitrification in controlling gaseous and hydrologic losses of nitrogen is also a major topic of study. Studies are ongoing in southern and central California and in the Mexico City Air Basin.
